# Molecular Interactions of Prodiginines with the BH3 Domain of Anti-Apoptotic Bcl-2 Family Members

**DOI:** 10.1371/journal.pone.0057562

**Published:** 2013-02-27

**Authors:** Ali Hosseini, Margarita Espona-Fiedler, Vanessa Soto-Cerrato, Roberto Quesada, Ricardo Pérez-Tomás, Victor Guallar

**Affiliations:** 1 Joint BSC-IRB Research Program in Computational Biology, Barcelona, Spain; 2 Cancer Cell Biology Research Group, Department of Pathology and Experimental Therapeutics, University of Barcelona, Barcelona, Spain; 3 Department of Chemistry, University of Burgos, Burgos, Spain; 4 Institució Catalana de Recerca i Estudis Avançats, Barcelona, Spain; University of Cincinnati College of Medicine, United States of America

## Abstract

Prodigiosin and obatoclax, members of the prodiginines family, are small molecules with anti-cancer properties that are currently under preclinical and clinical trials. The molecular target(s) of these agents, however, is an open question. Combining experimental and computational techniques we find that prodigiosin binds to the BH3 domain in some BCL-2 protein families, which play an important role in the apoptotic programmed cell death. In particular, our results indicate a large affinity of prodigiosin for MCL-1, an anti-apoptotic member of the BCL-2 family. In melanoma cells, we demonstrate that prodigiosin activates the mitochondrial apoptotic pathway by disrupting MCL-1/BAK complexes. Computer simulations with the PELE software allow the description of the induced fit process, obtaining a detailed atomic view of the molecular interactions. These results provide new data to understand the mechanism of action of these molecules, and assist in the development of more specific inhibitors of anti-apoptotic BCL-2 proteins.

## Introduction

**Figure 1:**
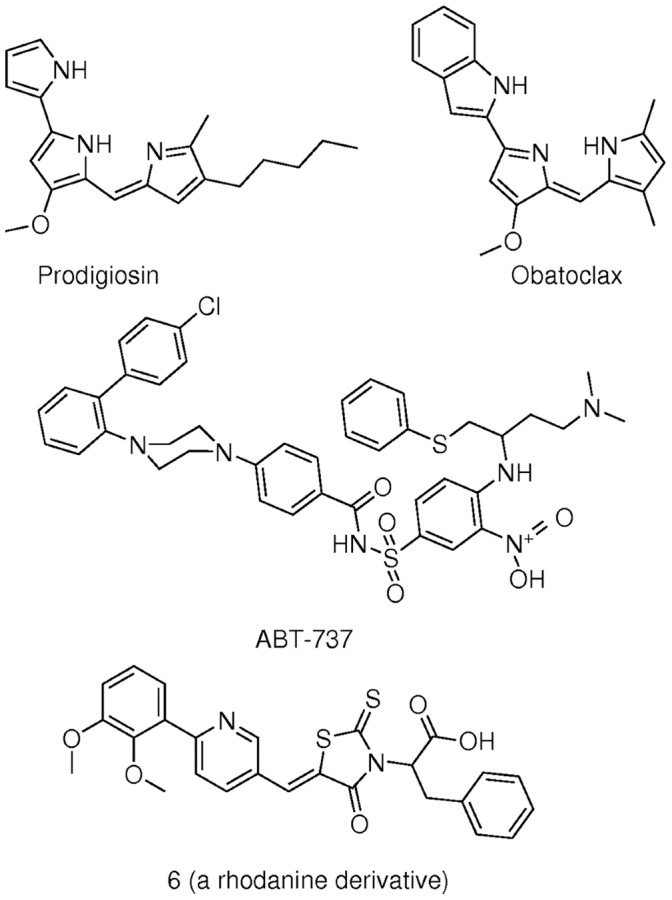
The ligands used. The ligands used in this study are shown: prodigiosin, obatoclax, ligand number 6 (a rhodanine derivative) and ABT-737.

In order to advance in molecular target therapies, it is important to elucidate the target and the atomic detailed mechanisms of protein-drug interactions. A wide set of experimental techniques, such as crystallography, NMR, calorimetry, etc, together with theoretical docking efforts aim to address this issue. Recently, we have turned our attention in solving the molecular target and the binding mechanism for prodigiosin (PG). PG, a bacterial metabolite from the prodiginine family (see Figure 1), has shown apoptotic activity against several cancer cell types with low cytotoxicity in non-malignant cells. The National Cancer Institute (dtp.nci.nih.gov) tested prodigiosin against a collection of ∼60 cancerous cell lines with an average half maximal inhibitory concentration (IC_50_) of 2.1 µM [1]. Furthermore, recent studies elucidated that PG triggers apoptosis by the intrinsic pathway [2], provoking the increase in the pro-apoptotic NAG-1 protein and the negative cell cycle regulator p21 [3], and inducing down-regulation of the inhibitor of apoptosis survivin [4], SKP2 [5] and RAD51 [6] proteins. Nevertheless, the direct molecular target(s) of this agent is still an open question.

Apoptosis, the programmed cell death that controls removal of damaged cells, is extremely well regulated by the extrinsic and intrinsic pathways. The key regulator proteins of the intrinsic pathway are known as the BCL-2 family, being BAX and BAK the pro-apoptotic members responsible for the mitochondrial outer membrane permeabilisation. Cytochrome c is then released to the cytosol allowing the activation of procaspase-9, leading to cell death [7]. In survival conditions, BAX and BAK are sequestered by the anti-apoptotic family members: BCL-2, BCL-X_L_, MCL-1, BCL-W, BCL-B and BCL2A1. These anti-apoptotic proteins share four regions of high sequence similarity known as the BCL-2 Homology (BH) domain, (BH1, BH2, BH3 and BH4). When a stress stimulus occurs, BH3-only proteins bind to the BH3 domain of anti-apoptotic BCL-2 proteins, displacing and releasing pro-apoptotic BAK or BAX, committing the cell to death. [7], [8].

BH3-mimetic molecules have emerged as promising anti-cancer drugs since they are able to directly reverse the evasion of apoptosis [9]. This is the case for Obatoclax (OBX), which binds to a broad spectrum of BCL-2 family members [10]–[13]. To elucidate whether PG also behaves as a BH3-mimetic drug and how these interactions occur at the molecular level, we combined again immunoprecipitation assays with *in silico* modeling [14]. We focused on MCL-1, BCL-xL and BCL-2, three anti-apoptotic proteins representative of selectivity patterns among BH3 domains within the BCL-2 family that have centered most of recent studies [15]–[17]. We find that PG antagonizes MCL-1 by binding to the BH3 domain triggering BAK release. Furthermore, we obtain an atomic detailed description of PG’s interaction in the BH3 domain. Altogether, these results show, for the first time, the BH3 mimetic nature of PG and provide a detailed atomic view of the molecular interactions of prodiginines (PGs) with the BH3 domain of several antiapoptotic BCL-2 proteins.

## Materials and Methods

### Reagents

Prodigiosin (2-methyl-3-pentyl-6-methoxyprodigiosene) was provided by Dr. R. J. Schultz of the National Cancer Drug Synthesis and Chemistry Branch Chemotherapeutic Agents Repository (Bethesda, MD). Obatoclax, a synthetic indol-containing prodiginine, was prepared by acid catalyzed condensation of 2-(4-methoxy-5-vinyl-1H-pyrrol-2-yl)-1H-indole and 2,4-dimethyl-1H-pyrrole [18]. All stock solutions were diluted in DMSO and stored at −20°C.

### Cell Lines and Culture Conditions

Human melanoma cancer cell line SK-MEL-5 was purchased from the American Type Culture Collection (Manassas, VA). Cells were cultured in Dulbeccós Modified Eaglés Medium (DMEM, Biological Industries, Beit Haemek, Israel) supplemented with 10% heat-inactivated foetal bovine serum (FBS; Life Technologies, Carlsbad, CA), 100 U/ml penicillin, 100 µg/ml streptomycin, and 2 mM L- glutamine, all from Biological Industries. Cells were grown at 37°C in a 5% CO_2_ atmosphere.

### Immunoblot Analysis

After their respective treatments, adherent and floating cells were lysed in immunoprecipitation (IP) buffer (50 mM Tris (pH 8.0), 60 mM KCl, 1 mM EDTA, 1 mM DTT, 0.5% Nonidet P-40 (IGEPAL), 10 mM sodium vanadate, 50 mM NaF, 1 µg/ml aprotinin, 1 µg/ml leupeptin, 1 µg/ml pepstatin and 0.1 mM PMSF) or lysis buffer (0.1% SDS, 1% NP-40, 0.5% sodium deoxycholate, 50 mM NaF, 40 mM β-glycerophosphate, 200 µM sodium orthovanadate, 1 mM phenylmethylsulfonyl fluoride and serine and cysteine protease inhibitor cocktail (Roche 11836170001)) for MCL-1 overexpression analysis. Total cell extracts were centrifuged at 12000×g for 10 min at 4°C. Protein concentration was determined with the BCA protein assay (Pierce, Rockford, IL) using bovine serum albumin as standard. 40 µg of protein extracts were separated by SDS-PAGE and transferred to Immobilon-P membranes (Millipore, Bedford, MA). They were then incubated with primary antibodies anti-MCL-1, BCL-2, BAK, BAX, and actin (Santa Cruz Biotechnology, Inc., Santa Cruz, CA), anti-Caspase- 9 and PARP (Cell Signalling, Beverly, MA) or anti-Vinculin (Sigma-Aldrich Chemical Co., St.Louis, MO) according to the manufacturer’s instructions. Antibody binding was detected with secondary antibodies conjugated to peroxidase and the ECL detection kit (Amersham, Buckinghamshire, UK).

### Co-immunoprecipitation from Cells

Cell extracts (800 µg of protein) were brought to a volume of 1 ml with IP buffer and incubated with 2 µg of anti-MCL-1 antibody (Santa Cruz Biotechnology), anti-BAK (Cell Signalling Technology) or anti-BAX antibody (Invitrogen, Carlsbad, CA) overnight at 4°C. Immune complexes were precipitated by incubation with protein A-coated agarose beads (Sigma-Aldrich Chemical Co.) previously equilibrated with IP buffer, and washed three times with 0.5 ml of IP buffer. Immunoprecipitated proteins were loaded on a 12% SDS-PAGE gel and analyzed by Immunoblot using anti-MCL-1, anti-BAK and anti-BAX antibodies (Santa Cruz Biotechnology).

### MCL-1 Overexpression and Cell Viability Evaluation

SK-MEL-5 cells were seeded in 6-well plates and allowed to grow up to 70% confluence. Before transfection, growth media was replaced by Optimem media without FBS (Invitrogen) and 1 µg of plasmidic DNA was transfected to cells using 20 µl of lipofectin reagent (Invitrogen) per condition. Empty pcDNA3-GFP vector or pTOPOMCL1 plasmid (Addgene plasmid 21605 [19], were used. After 20 h of transfection, the media was replaced by complete media with or without 2 or 20 µM PG or OBX, respectively. After 24 h of treatment, cells were resuspended and 100 µl of each condition were passed in triplicate to a 96-well plate. Cell viability was determined using the methyl-thiazole-tetrazolium (MTT) assay [20]. Briefly, 10 µM of MTT (Sigma Chemical Co.) was added to each well for an additional 2 h. The blue MTT formazan precipitate was dissolved in 100 µl of isopropanol: 1N HCl (24∶1). The absorbance at 570 nm was measured on a multiwell plate reader. Cell viability was expressed as a percentage of non-treated cells and data are shown as the mean value ± S.D. of two independent experiments. Statistical analysis (ANOVA and LSD tests) was carried out with the STATGRAPHICS Centurion XVI.I. statistical package. *P*<0.05 and *P*<0.01 were represented with * and **, respectively. To determine transfection efficiency, immunoblot analyses were performed to assess MCL1 protein expression levels in each condition.

### Analyses with Isolated Mitochondria

SK-MEL-5 cells were treated with PG (100 nM), OBX (10 µM) or DMSO (vehicle) at 37°C for 24 h. Cells were lysed in ice-cold 25 mM Tris (pH 6.8), 250 mM sucrose, 1 mM EDTA, 0.05% digitonin, 1 mM DTT, 1 µg/ml aprotinin, 1 µg/ml leupeptin, 1 µg/ml pepstatin, 0.1 mM PMSF. Samples were then centrifuged at 13000×g for 5 min at 4°C. Mitochondrial fraction (pellet) was isolated, washed once and resuspended with lysis buffer. Total cell lysate, mitochondrial and cytosolic fractions were analyzed by Immunoblot using cytochrome c, porin and actin antibodies (Santa Cruz Biotechnology).

### Computational Methods

Computational docking was modeled by combining PELE (Protein Energy Landscape Exploration) [21] with Glide [22]. To map protein-ligand conformational changes and induced fit we used our in house program PELE, a Monte Carlo algorithm where new trial configurations are produced with sequential ligand (and protein) perturbation, side chain prediction and minimization steps. Ligand perturbation includes a ligand specific rotamer library. Trial configurations are then filtered with a Metropolis acceptance test, where the energy is described with an all-atom OPLS force field with a surface generalized Born solvent model. PELE has recently shown to provide more accurate induced fit results than the state of the art commercial software [23], and to reproduce the conformational sampling obtained in microsecond molecular dynamics trajectories with two orders of magnitude reduction in computational cost [24].

We have modeled PG and OBX binding to three different anti-apoptotic BCL-2 members for which crystal structures and inhibitors (as controls) are known: MCL-1, BCL-2 and BCL-xl.

#### MCL-1

To model MCL-1 we used the crystal structure bound to a BH3-peptide, pdb code 2NLA [25]. For the control simulation we used the ligand named 6, a derivative of rhodanine that has and IC50 of 0.25 µM [26]. Additionally, OBX has been shown to bind to MCL-1 with an IC50 of 2.9 µM. [10]–[13].

#### BCL-2

To model BCL-2 the crystal structure bound to the 43B peptide (BH3 mimic), pdb code 1YSW was used [27]. For the control simulation we used the ligand ABT-737, with an IC50 of 0.12 µM [10], [28].

#### BCL-xL

To model BCL-xL we used a crystal structure bound to a known inhibitor, ABT-737, with pdb code 2YXJ [28], [29]. For the control simulation we used the ligand present in the initial crystal, ABT-737, with an IC50 of 0.06 µM [10], [29].

All ligands used in this study are shown in Figure 1.

For all systems we removed the crystallographic ligands and prepared the protein with Schrodinger’s Protein Wizard [30]. This algorithm builds hydrogen-bonded clusters and performs 100000 Monte Carlo moves by reorienting hydroxyl and thiol groups, water molecules, amide groups of Asn and Gln, and the imidazole ring in His. The algorithm also predicts protonation states of His, Asp, Glu, Lys and Arg. Each possibility is scored based on the total number of hydrogen bonds and their quality (relative to an idealized hydrogen bond). In particular, all Asp, Glu, Lys and Arg kept their anionic state. Histidines 224, 252 and 277 in MCL-1, and 117 and 183 in BCL-2 were epsilon protonated; all other histidines kept the default delta protonation. The ligand’s atomic charges were derived from the electrostatic potential fitting of a single point DFT/B3LYP calculation with the 6-31G** basis set.

#### Ligand docking and induced fit procedure

After the protein and ligand’s preparations, we performed a cavity search with SiteMap [31], which confirmed the BH3 domain as the top ranked binding cavity in the three systems. Initial rigid docking was performed with Glide [22] using the extra precision (XP) scoring function [32], currently viewed as the state of the art docking software/procedure. Correlation of Glide scores with binding affinities, however, can only be done at a qualitative level. While score values below −8/−9 indicate a good binder, a more quantitative assessment requires a system specific control. Thus, for each protein we docked an inhibitor with known (good) binding affinities. Comparing the values predicted for these control ligands with those obtained for PG, we could estimate more accurately their binding strength.

Following the rigid docking we performed 8 independent PELE trajectories, each including 600 iterations (24 hours), of induced fitted adjustment. We then clustered the trajectory in 5 groups, based on the ligand’s heavy atom root mean square deviation (RMSD), and selected the median in each group. For each representative cluster we re-docked all ligands with Glide. Thus, for each system and ligand we have two XP score values: an initial one biased to the crystal structure and a final score after the induced fit (the largest score from the 5 clusters), which aims to adapt the protein to each specific ligand.

## Results

### PG Disrupts MCL-1/BAK Complex in Melanoma Cells

**Figure 2:**
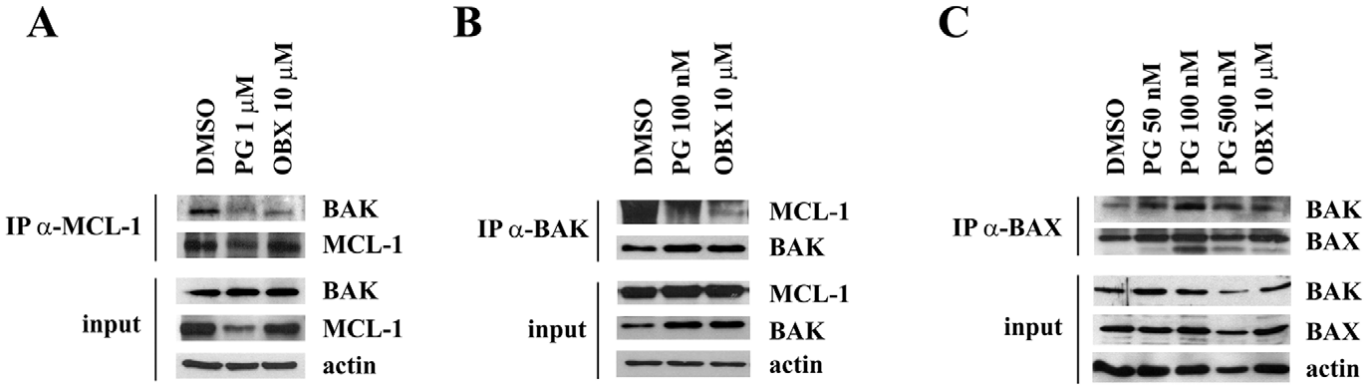
PG is a BH3-mimetic molecule. (**A** and **B**) PG disrupts constitutive MCL-1/BAK interaction. Cell lysates were subjected to immunoprecipitation with anti-MCL-1 or anti-BAK antibody after PG treatment at 1 µM (8 h) and 100 nM (24 h), respectively. (**C**) **PG permeabilizes the outer mitochondrial membrane.** BAX was immunoprecipitated from PG (50, 100 and 500 nM), OBX (10 µM) or DMSO- treated cells for 24 h and then BAK/BAX complex formation was analyzed by immunoblot with anti-BAK and anti-BAX antibodies.

BCL-2 family member MCL-1 binds to and regulates BAK within the mitochondrial outer membrane [33] and the BH3 mimetic molecule OBX has been reported to inhibit this constitutive interaction [34]. To examine whether PG could also alter this binding, MCL-1/BAK complex was co-immunoprecipitated from PG, OBX or DMSO- treated SK-MEL-5 cells. In DMSO-treated cells, MCL-1 was co-immunoprecipitated with BAK using MCL-1 antibody, indicating that these proteins heterodimerize in basal conditions. Alternatively, treatment with PG or OBX resulted in a complete release of BAK from MCL-1, showing the BH3 mimetic nature of PGs (Figure 2A). As MCL-1 levels were downregulated by 1 µM PG, lower doses were used to corroborate these results. Similarly, BAK immunoprecipitation resulted in the appearance of MCL-1/BAK complex in non-treated cells, whilst this binding disappeared in treated cells and no protein level modifications were observed (Figure 2B). Finally, since BAK and BAX heterodimerize when they are released from their anti-apoptotic proteins MCL-1 and BCL-2 (in order to form pores in the mitochondrial membrane), the formation of this complex was analyzed in treated cells. As observed in Figure 2C, after 24 h of treatment, PG as well as OBX induced BAK/BAX complex formation, indicating mitochondrial membrane permeabilization.

### Mitochondrial Apoptosis is Triggered after PGs Treatment

**Figure 3:**
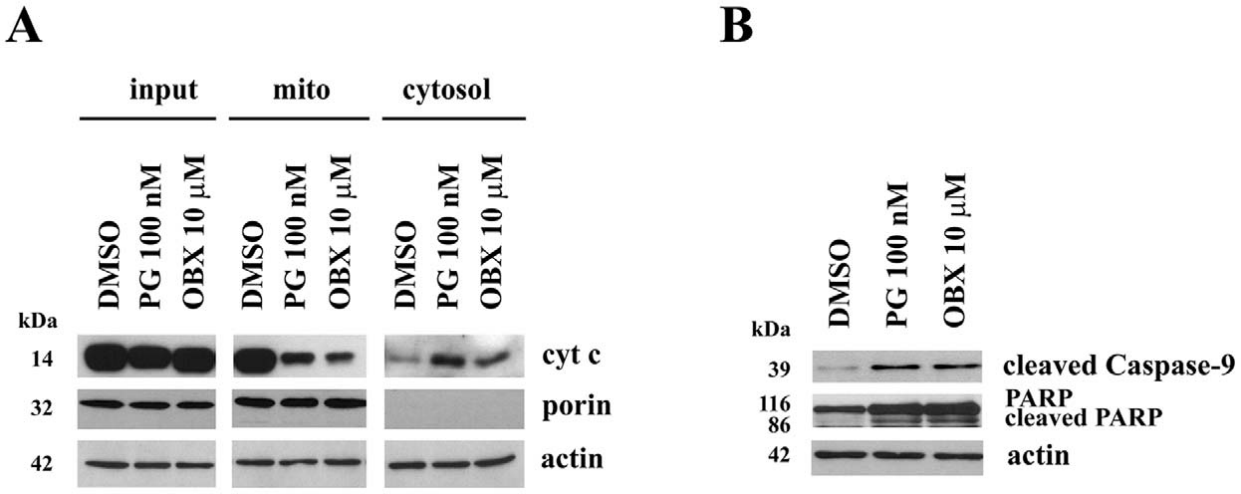
PG induces activation of the intrinsic apoptotic pathway. (**A**) PG induces cytochrome c release to the cytosol. Cytochrome c release from SK-MEL-5 isolated mitochondria after PG and OBX treatment. Mitochondria were isolated from PG (100 nM), OBX (10 µM) or DMSO- treated cells for 24 h. Cytochrome c release from mitochondria to cytosol was analyzed by Immunoblot using the mitochondrial marker porin as a quality control of the isolation process. (**B**) **Activation of caspases.** Cells were treated with PG (100 nM) or OBX (10 µM) for 24 h and total cell lysates were analyzed by immunoblot. Actin was used as loading control.

At the same time that MCL-1/BAK complex was disrupted by PGs treatment and the mitochondrial membrane pore was formed, we observed cytochrome c release from the mitochondria to the cytosol (Figure 3A). This protein binds to Apaf-1 and caspase-9 to form the apoptosome and this complex facilitates caspase-9 activation by proteolysis. In Figure 3B we can observe cleaved caspase-9 appearance after PGs treatment, indicating its activation. Moreover, we also observe cleavage of the caspase substrate called PARP, corroborating caspases activation. Altogether these results demonstrate the activation of the intrinsic apoptotic pathway after PGs treatment.

### MCL-1 Overexpression Partially Abrogates PGs Induced Cell Death

**Figure 4:**
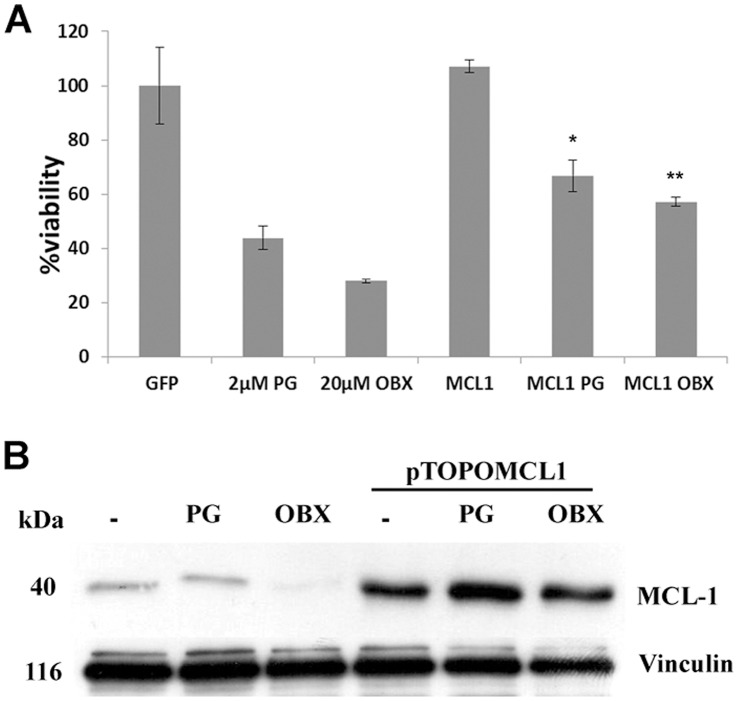
Effect of MCL-1 overexpression on cell viability. (**A**) MCL-1 overexpression partially blocks PGs cytotoxicity. SK-MEL-5 cells were transfected with 1 µg of pTOPOMCL1 plasmid and, after 20 h, cells were treated with PGs (2 and 20 µM, respectively) for an addittional 24 h. Then cell viability was assessed by the MTT assay. (**B**) **Analysis of MCL-1 protein levels.** After MCL1 overexpression and PGs treatment, MCL-1 protein levels were analyzed by Immunoblot. Vinculin was used as a loading control.

To elucidate whether MCL-1 was involved in the cytotoxic effect triggered by PGs, MCL-1 was overexpressed in SK-MEL-5 cells. After 20 h from transfection, PGs treatment was added during 24 h and cell viability was analyzed by MTT assay (Figure 4). PGs-induced cytotoxic effect was significantly blocked by overexpressing MCL-1. These results might suggest that PGs are not able to disrupt all the MCL-1/BAK complexes when MCL-1 is overexpressed, preventing some BAK protein to form mitochondrial membrane pores, though being lower the apoptotic effect. Figure 4 B shows MCL-1 protein levels at basal or overexpressing conditions after PGs treatment.

### Molecular Docking

Table 1 summarizes the docking results for MCL-1, BCL-xL, and BCL-2. Besides from PG and OBX, for each anti-apoptotic target we have docked a control ligand known to be a good binder. For each ligand and target we show the initial docking score, biased towards the initial (crystal) structure, and the final score as a results of the induced fit (after PELE simulation). For MCL-1 the initial docking was performed after removing the BH3 peptide from the crystal structure. Thus, we expect a significant RMSD change and an improvement in the docking score along the induced fit process. Clearly for all ligands we observe a large RMSD, ranging from 3 to 7, and a significant increase in the score. Interestingly, similar scores (∼−9) are obtained for the control, ligand 6, and for the two prodiginines under study, OBX and PG.

**Table 1:** Before and after induced fit docking scores.

	MCL-1			BCL-xL			BCL-2		
Ligands	PG	OBX	6	PG	OBX	ABT	PG	OBX	ABT
Initial Score	−4.3	−2.9	−6.0	−7.0	−3.4	−7.9	−6.3	−6.0	−8.5
Final Score	−8.6	−8.8	−8.7	−7.4	−8.3	−13.9	−7.9	−8.8	−9.6
RMSD	3.0	4.0	7.4	3.0	5.5	1.4	4.8	4.5	4.0

Also shown is the ligand RMSD along the induced fit process.

For BCL-xL the initial crystal structure used to model the target already has the control ligand ABT-737 bound to it. Thus, as expected, we observe the lowest induced fit RMSD for this ligand, 1.4 Å. Additionally, we find good initial and (very good) final scores for ABT-737 a potent inhibitor of BCL-xL from Abbott Laboratories with an IC50 of 0.06 µM [10]. For OBX we observe 5.5 Å RMSD change along the PELE simulation, together with a large improvement of its binding score. For PG, we observe a lower final score and a medium induced fit, pointing to micromolar rather than nanomolar activity.

In BCL-2 our model was derived from a peptide (43B) bound crystallographic structure. Accordingly, we observe again significant induced fit RMSD changes and improvements in the scores. For ABT-737 we observe good initial scores and the lower RMSD, possibly as a result of its large size and excellent BH3 helix mimetic properties. As expected from its IC50 of 0.12 µM [10], the final score is −9.6. OBX is again the second best scorer followed by PG.

**Figure 5:**
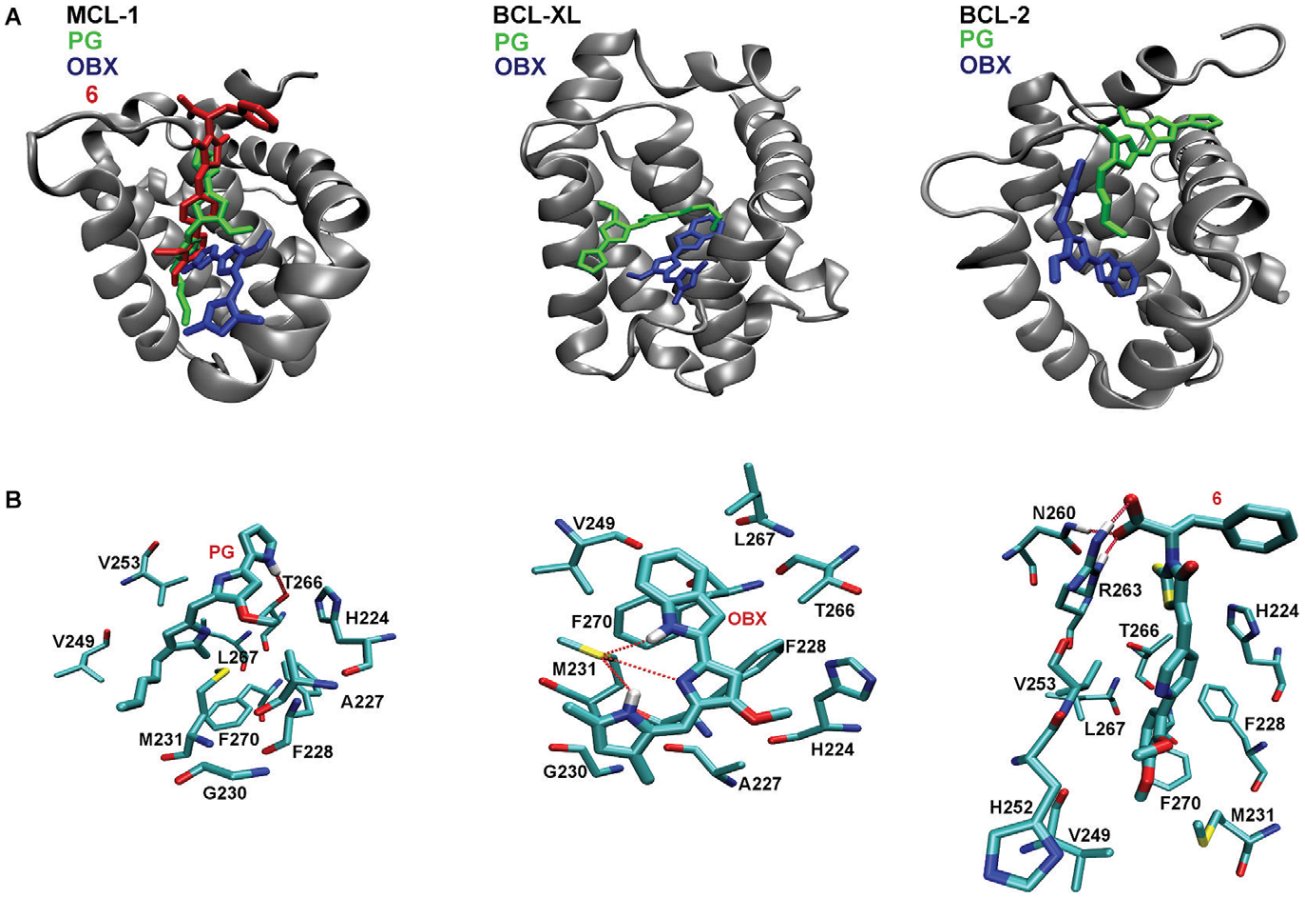
Induced fit docked structures. Top panels (A): final induce fit structures in MCL-1 (left) BCL-xl (center) and BCL-2 (right). Bottom panles (B): detailed view of the molecular interactions of PG, OBX and 6 with MCL-1.

Figure 5 shows the induced fit structures obtained after modeling with PELE. The left panel in Figure 5A compares the final structures for OBX, PG and 6 in the BH3 binding domain of MCL-1. The center and right panels show the final structures obtained for OBX and PG in BCL-xL and BCL-2. For simplicity, we superimpose the final position for each ligand into a consensus ribbon representation. In all three proteins, PG and OBX use slightly different regions of the BH3 domain and present different protein-ligand interactions. Figure 5B shows the protein-ligand interactions for PG, OBX and 6 with MCL-1. All three ligands bind to the hydrophobic core defined by Val253, Val249, Ala227 and Leu267. PG forms a hydrogen bond with Thr266, OBX makes a hydrogen bond with Met231 and ligand 6 makes hydrogen bonds with Asn260 and Arg263. Despite sharing a similar molecular skeleton, PG and OBX present important differences in the hydrogen bond network. As seen in Figure 1, a hydrogen bond acceptor ring in PG turns into a hydrogen bond donor in OBX, explaining the differences in the binding modes. In supporting information we provide a pharmacophore analysis (Supplementary Figure S1) and the atomic coordinates (in pdb format) for the docked structures shown in Figure 5A, the best scoring structures for each ligand and protein.

## Discussion

The proapoptotic agent prodigiosin has shown an average IC_50_ value of 2.1 µM when tested against a collection of ∼60 cancerous cell lines [1]. Thus, it seems a good candidate as a base drug to carry on further development. For this, it is necessary to elucidate its target(s) and obtain an atomic detailed description of its binding mechanism. In a previous study we analyzed the interactions of PG and OBX with several kinases, demonstrating that the mammalian target of rapamycin (mTOR) is a molecular target of both PGs in melanoma [14]. Moreover, these results also showed that inhibition of mTOR was accompanied by the activation of both cell death mechanisms, autophagy and apoptosis. Here we show how PG, as indicated previously for OBX [10], binds to the BH3 domain of the anti-apoptotic BCL-2 family, synergizing the proapoptotic effects induced by mTOR inhibition. In particular, in melanoma cells, we demonstrate that PG disrupts the interaction between MCL-1 and BAK, allowing the formation of BAK/BAX complex and the subsequent cytochrome c release to the cytosol, which mediates the mitochondrial apoptosis activation. Moreover, MCL-1 has been identified as a molecular target directly involved in PGs induced cell death, since its overexpression is able to decrease PGs cytotoxicity.

In order to characterize the protein-drug complex, we used recent advances in protein-ligand recognition software. It is clear from the results that the induced fit process is essential in order to get good binding affinities. Rigid docking into the crystals, for example, would fail to recognize ligand 6 as a nanomolar binder in MCL-1. Furthermore, the induced fit simulations allow comparing the ABT-737 scores with the experimental IC_50_ in BCL-xL and BCL-2. Overall, the results with the two prodiginines and the three BCL-2 family members indicate good protein-ligand interactions when compared to control ligands. In MCL-1, in particular, PG scores are similar to the control ligand, suggesting an IC_50_ in the hundreds of nanomolar range.

**Figure 6:**
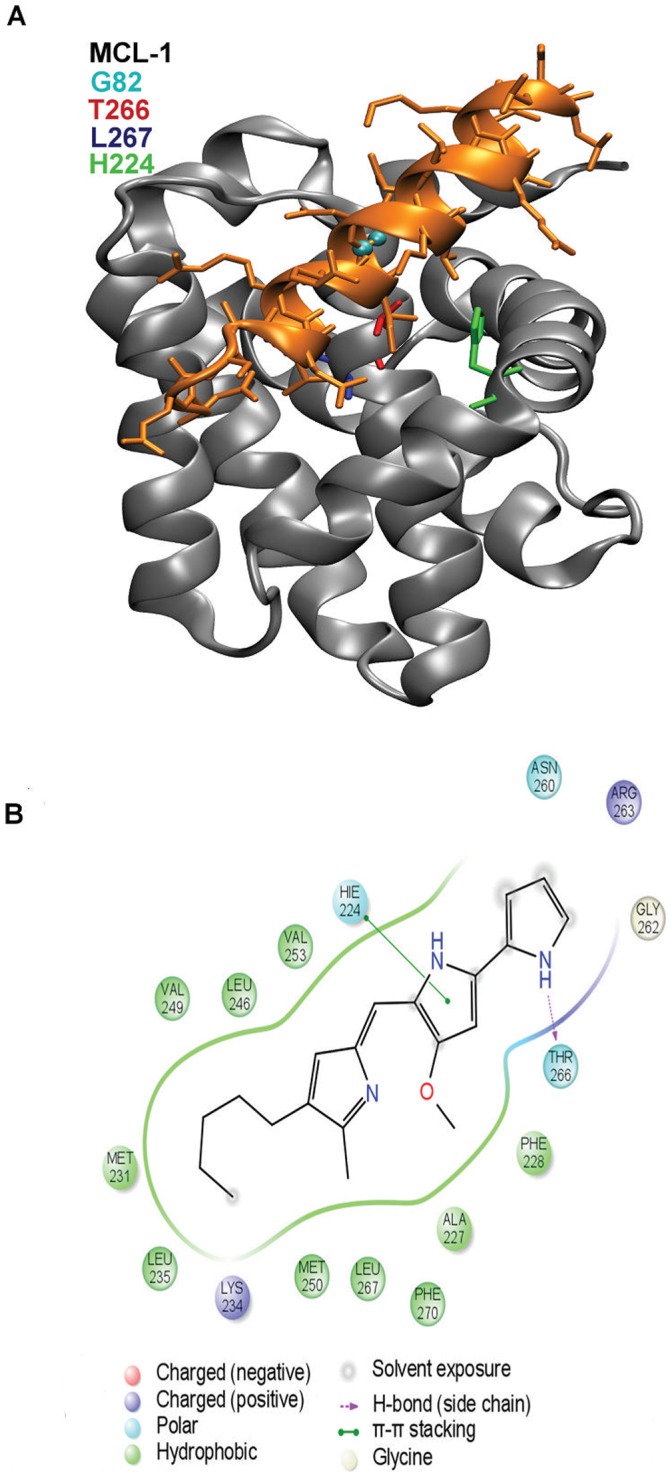
MCL-1 contact map with PG. (A) Crystal structure of the mNoxaB BH3 peptide bound to MCL-1. In cyan we show Gly82, the 11 helix residue located in the h3 position. Leu267 in blue, Thr266 in red and His224 in green. from MCL-1 are also shown. (B) Pharmacophore analysis of the binding interactions. Residues within 3 Å from the ligand have been included in the anlysis.

PG, OBX and ligand 6 bind in a specific region of the MCL-1 BH3 domain, defined by a hydrophobic core including Val253, Val249, Ala227 and Leu267. This region corresponds closely to the h1–h3 position of the BH3 peptides. Figure 6A shows the interaction of a BH3 helix peptide with MCL-1. Peptide residue 11, located at the h3 position of the helix (G82 in the crystal structure, shown in cyan), is a conserved small residue in contact with the hydrophobic core, which has been shown to be important for ligand binding [35], [36]. Additional studies by Chen et al. [15] and Day et al. [37] have also underlined the importance of positions h1 and h3 for binding to MCL-1 and BCL-xL. Computational studies also pointed to this hydrophobic core in the MCL-1 ligand recognition [26], [38]. Interestingly, in BCL-xL, the two prodiginines have more distant binding modes, centered in the h1–h2 region, in agreement with the larger degree of flexibility observed in this end of the helix for BCL-xL [35], [36].

Figure 6B shows a pharmacophore analysis for the PG/MCL-1 binding interactions (the other ligand and proteins are shown in supporting information). Clearly, the combination of the hydrophobic interactions (in green) together with the hydrogen bond to Thr266 play a key role in PG’s binding. Stacking to His224 is also underlined in the pharmacophore analysis [15]. To further analyze the role of Thr266, we have modeled a single mutation T266A in the active site of MCl-1. The final score, associated with a 4.2Å RMSD, is reduced to −6.3 (instead of a −8.6 for the wild type), indicating the importance of this residue for binding. Obviously, confirmation of these binding modes simulations and residue analysis will require future directed mutagenesis.

The identification of PG as a new BH3 mimetic molecule, together with previous results demonstrating the potential of PG as an inhibitor for both mTOR complexes [14], evidences the potential of PG as a chemotherapeutic agent. In fact, emergent molecular therapies are focused on molecules that are able to target multiple proteins involved in cell survival. Molecules such as PP242 (ATP mimetic inhibitor) [39] or ABT-263 (analog of ABT-737) [40] have shown similar successful results as OBX in clinical trials [11], [34]. Nevertheless, combinational strategies are still necessary to improve the effect of these molecules. Based on previous results which markedly enhanced OBX-mediated cell death [12], [13], we might consider combining PG with the ER stress inducers such as tunicamycin [41], cisplatin [42] or sorafenib [43], [44] which markedly enhanced apoptotic cell death.

Altogether, our results demonstrate, for the first time, that MCL-1 is a molecular target of PG involved in its cytotoxic effect and that this is due to the capacity of PG to displace activating BH3 proteins from the pocket of MCL-1 triggering BAK oligomerization and the subsequent cytochrome c release-mediated apoptosis.

### Supporting Information

The coordinates (in pdb format) and pharmacophore analyses are provided for the best scoring structures for each ligand and protein.

## Supporting Information

Figure S1 **Pharmacophore analysis.** Pharmacophore analysis for the binding interactions of all residues with all proteins. Residues within 3 Å from the ligand have been included in the anlysis.
(PDF)

File S1 **The coordinates (in pdb format) are provided for the best scoring structures for each ligand and protein.**
(PDB)
