# Supplementary material for: Molecular Interactions of Prodiginines with the BH3 Domain of Anti-Apoptotic Bcl-2 Family Members
Source: PLoS One. 2013 Feb 27;8(2):e57562. doi: 10.1371/journal.pone.0057562 (PMC3583838; doi:10.1371/journal.pone.0057562)
Supplement: Figure S1 — Pharmacophore analysis. Pharmacophore analysis for the binding interactions of all residues with all proteins. Residues within 3 Å from the ligand have been included in the anlysis. (PDF) [file pone.0057562.s001.pdf]

Pharmacophore analysis of the binding interactions.

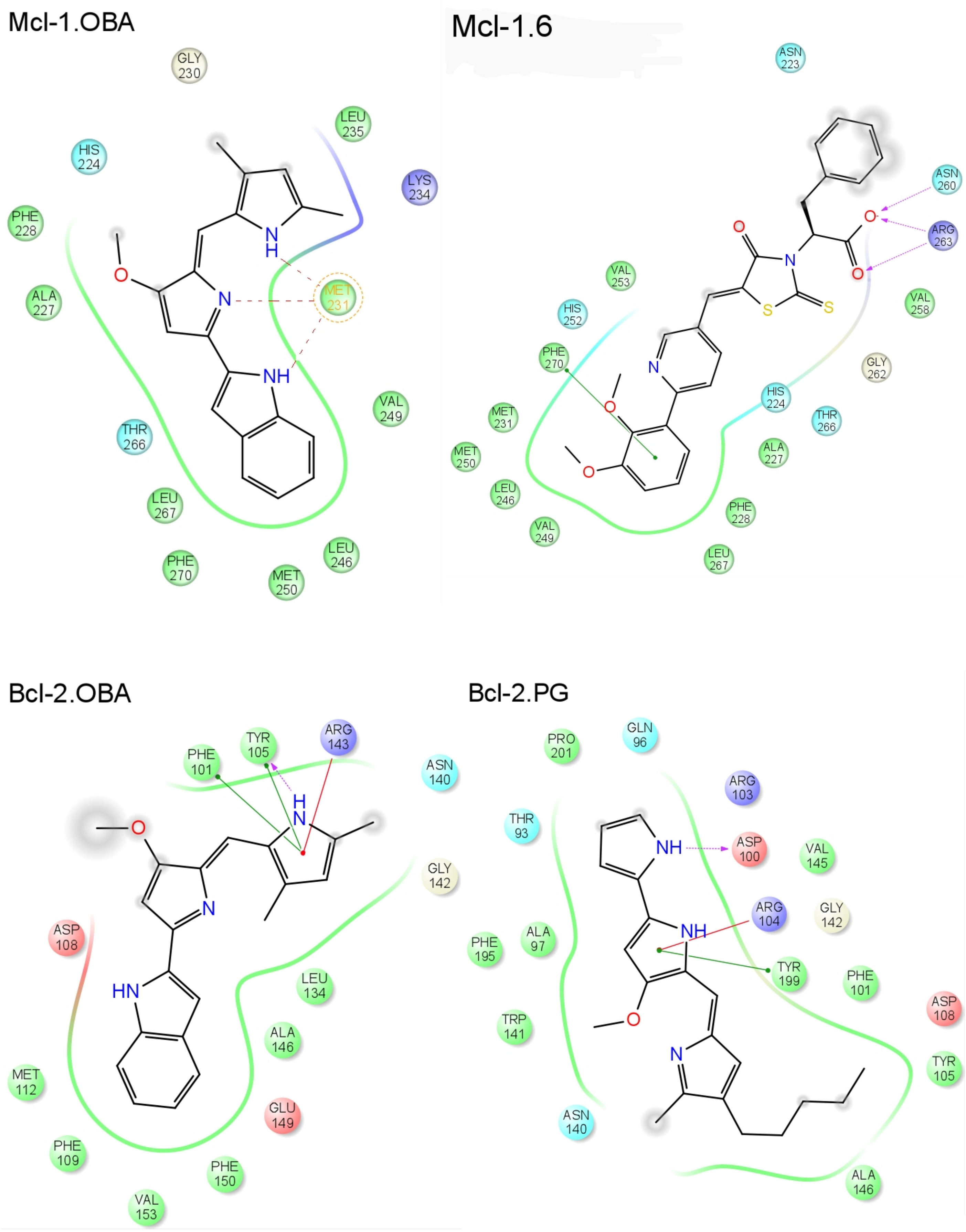

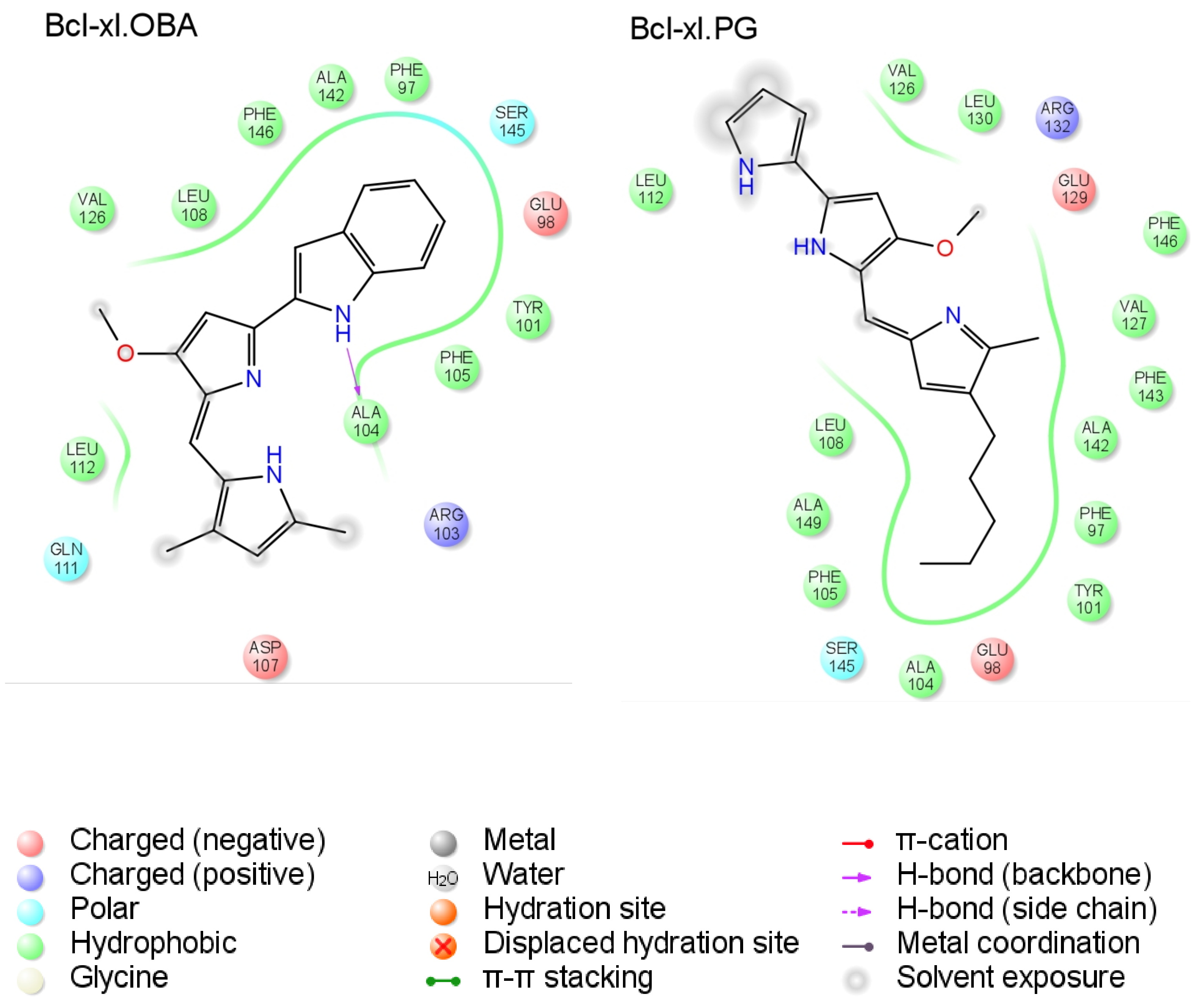
Bcl-xl.OBA
Bcl-xl.PG
PHE 97
ALA 142
PHE 146
SER 145
VAL 126
LEU 108
GLU 98
TYR 101
PHE 105
ALA 104
LEU 112
ARG 103
GLN 111
ASP 107
VAL 126
LEU 130
ARG 132
LEU 112
GLU 129
PHE 146
VAL 127
PHE 143
LEU 108
ALA 142
ALA 149
PHE 97
PHE 105
TYR 101
SER 145
ALA 104
GLU 98
Charged (negative)
Charged (positive)
Polar
Hydrophobic
Glycine
Metal
H2O Water
Hydration site
Displaced hydration site
π-π stacking
π-cation
H-bond (backbone)
H-bond (side chain)
Metal coordination
Solvent exposure
